# Mr. Stables, on the Influenza

**Published:** 1803-10-01

**Authors:** J. Stables

**Affiliations:** Storsforth, Yorkshire


### *To the Editors of the Medical and Physical Journal.*

GENTLEMEN,

THE late prevailing epidemic, *called the Influenza,* made its appearance in our neighbourhood in the month of April; but in the two following months, May and June, it was by far the most prevalent. I have also seen several well marked cases of it so late as the latter end of August: from the observations I made of it, I am induced to think it not contagious. My own servant boy, the latter end of June, felt the effects of it very severely for about ten days; the remainder of the family, eight in number, (though no precaution was observed to prevent intercourse) did not apparently receive the least inconvenience from it. From which, and several similar instances, I had strong reasons to suppose its contagion atmospheric, and not communicable from person to person. The epidemic shewed itself both pneumonic and of the low typhoid kind; *but the latter* was the most prevalent form of it: the medicines which I made use of in general were the aq. amon. acet. *or the alkal. volatil.* well saturated with lemon juice. As to antimonials, in a few cases which I thought strongly indicated the use of them, and which, in similar cases, I had seen good effects from, evidently did harm. Opiates, particularly when the disease pertained to the typhoid kind, I found pleasing effects from. The few cases which proved fatal under my care were taken off between the fourth and the seventh day.

I am, &c.

*Storsforth, Yorkshire,*

*Sept.* 11, 1803.

J. STABLES.

